# Three‐year outcomes of childhood inflammatory bowel disease in New Zealand: A population‐based cohort study

**DOI:** 10.1002/jgh3.12310

**Published:** 2020-02-07

**Authors:** Natalie G Martin, Amin J Roberts, Helen M Evans, Jonathan Bishop, Andrew S Day

**Affiliations:** ^1^ Department of Paediatrics University of Otago Christchurch Christchurch New Zealand; ^2^ Starship Child Health Starship Children's Hospital Auckland New Zealand

**Keywords:** Crohn's disease, disease activity, treatment, ulcerative colitis

## Abstract

**Background and Aim:**

High rates of inflammatory bowel disease (IBD) have been documented in New Zealand (NZ) children. The objectives of this study were to describe the outcomes and disease course of childhood IBD in the first 3 years following diagnosis.

**Methods:**

All children diagnosed with IBD in 2015 in NZ were included. Clinical data obtained during routine care for 3 years following diagnosis were analyzed. Growth parameters, disease activity scores, and blood parameters were compared at diagnosis and follow up.

**Results:**

Three‐year outcome data were available for 48 of 51 children. At follow up, median age was 15.1 years, and 34 had Crohn's disease (CD), 11 had ulcerative colitis (UC), and three had IBD‐unclassified (IBDU). Although disease progression including development of perianal disease occurred in 13 (38%) of 34 children with CD, the majority (*n* = 30) had inflammatory disease at follow up. Disease extension occurred in 25% (2/8) of children initially diagnosed with UC. Of all IBD patients, the mean body mass index z‐score increased from −0.40 to +0.10 (*P* = 0.01). Disease activity scores reduced from diagnosis to follow up in both CD (mean pediatric Crohn's disease activity index 35–6, *P* < 0.001) and UC (mean pediatric ulcerative colitis activity index 44–6, *P* < 0.001). Overall, 56% of children received steroids, 44% of children with CD received biologic therapy, and four children with CD or UC had intestinal surgery.

**Conclusions:**

Most children with IBD were in remission with improved growth 3 years after diagnosis. Biologic therapies were commonly prescribed. This is the first NZ study assessing disease course in pediatric IBD. Ongoing follow up will continue to inform outcomes.

## Graphical abstract

This manuscript aimed, for the first time, to assess and describe the outcomes of a cohort of New Zealand children diagnosed with inflammatory bowel disease. Three years after diagnosis, this group of children was predominantly in remission with satisfactory improvements in growth parameters.

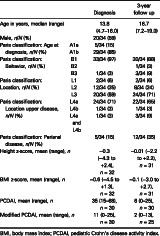

## Introduction

High rates of inflammatory bowel disease (IBD) have been documented in New Zealand (NZ) children, with a fourfold increase in incidence reported in the Canterbury region of NZ from 1996 to 2015.^1^ The incidence of pediatric IBD across NZ, as reported by a prospective study in 2015, was 5.2/100 000 for any IBD, 3.5/100 000 for Crohn's disease (CD), and 1/100 000 for ulcerative colitis (UC).^2^ These NZ rates were comparable to reported IBD rates in Europe.^3^, ^4^, ^5^ Previous studies of pediatric IBD in NZ have found a predominance of CD.^1^, ^2^, ^6^ Although IBD incidence peaks in adults, pediatric onset disease is reported in around one quarter of cases,^7^, ^8^ which may relate to early exposure to environmental risk factors or a greater number of genes conferring susceptibility.^9^ Childhood‐onset IBD has been associated with more severe disease and more extensive disease distribution in both CD and UC.^10^, ^11^, ^12^

Previous international studies have assessed disease characteristics and outcomes of children with IBD,^12^, ^13^, ^14^, ^15^, ^16^, ^17^, ^18^, ^19^, ^20^, ^21^ including reporting results of the widely accepted Paris classification to assess phenotype.^22^, ^23^ A previous study reported that complicated disease at diagnosis was associated with more surgery and hospitalizations in CD, and more extensive disease at diagnosis was associated with more biologic use in UC.^13^ Isolated colonic CD has been associated with a decreased risk of surgery.^13^, ^14^

In the 2015 prospective Pediatric Inflammatory Bowel Disease in New Zealand cohort study (PINZ study), 51 NZ children were diagnosed with IBD.^2^ No previous study has assessed outcomes of childhood IBD in NZ children. This study aimed to assess disease characteristics and outcomes in children recruited to the PINZ study at 3 years following diagnosis.

## Methods

### *Study population*

Every child aged <16 years diagnosed with IBD and residing in NZ between 1 January and 31 December 2015 was recruited to the PINZ study.^2^ Cases were identified prospectively by pediatric gastroenterologists, who provide care for all children with IBD in NZ. In addition, members of the NZ Society of Gastroenterology and private adult gastroenterologists were asked to provide details of any new pediatric IBD cases. IBD was diagnosed using standard criteria.^23^ The 51 children, including 34 children with CD, 10 with UC, and 7 with inflammatory bowel disease‐unclassified (IBDU), recruited to the PINZ study^2^ were eligible for inclusion in this retrospective follow‐up study.

### *Disease classification*

The phenotype and disease behavior of all subjects were assessed using the Paris classification, developed in 2011 as a modification for children of the Montreal classification.^22^

### *Disease activity scores*

A well‐established tool for analyzing disease activity in childhood CD is the pediatric Crohn's disease activity index (PCDAI), which includes assessment of symptoms, growth parameters, and laboratory results including inflammatory markers, with a maximum score of 100 (highest disease activity).^24^ A modification of the PCDAI (mod PCDAI) score is limited to laboratory results, including inflammatory markers, with a maximum score of 25.^24^ The pediatric ulcerative colitis activity index (PUCAI) measures disease activity by assessing gastrointestinal symptoms and activity restriction, with a maximum score of 85.^25^

### *Data collection and analysis*

Clinical data obtained during routine care were collected in 2018 and during the three intervening years since diagnosis. Data were collected by two pediatricians using a standardized case report form. All hospital clinic letters, admissions, investigations, and prescriptions were reviewed.

Demographic data were analyzed. Disease course was described including progression of Paris classification,^22^ perianal disease, disease flares, hospitalizations, and extraintestinal manifestations (EIM). The Paris classification was also described separately in children first diagnosed with CD at age <10 years. Clinical characteristics were compared at diagnosis and most recent follow up, including height z‐score, body mass index (BMI) z‐score, PCDAI score, modified PCDAI score, and PUCAI score. Inflammatory markers and blood parameters were also compared at diagnosis and most recent follow up separately for all IBD and CD only patients. Disease management was described, including corticosteroid management, biologic therapy, immunomodulatory therapy, oral and rectal 5‐aminosalicylic acid (ASA), antibiotic courses, and exclusive enteral nutrition (EEN) at diagnosis and subsequently. The cumulative steroid dose was calculated by converting each course of steroids to prednisolone equivalents per kilogram weight and summing all courses of steroids. Surgical management and treatment for iron deficiency were also described.

### *Statistical methods*

Statistical analysis was performed using IBM SPSS Statistics for Windows, version 25 (Armonk, NY, USA). The paired sample *t*‐test was used to compare continuous parametric variables, and the related‐samples Wilcoxon Ranked Sign test was used to compare continuous nonparametric variables. Fisher's exact test was used to compare categorical variables. Children with missing data were excluded pairwise. A *P* value of <0.05 was considered significant.

### *Ethical considerations*

Ethical approval was obtained from the University of Otago Human Research Ethics Committee reference HD18/034.

## Results

### *Demographics*

Of 51 children aged <16 years diagnosed with IBD in NZ in 2015, outcome data were available for 48 children at a mean of 3.1 years following diagnosis. The median age at diagnosis was 12.3 years (range 1.0–15.9 years), and 58% (28/48) were male. Children living in the South Island comprised 52% (25/48) of participants, with 48% living in the North Island of NZ. Three participants in the 2015 PINZ cohort study were lost to follow up, including one child with IBDU and two with UC.

Of 48 included children, 34 had CD, 11 had UC including 3 who were initially diagnosed with IBDU, and 3 had IBDU. Of children with IBD residing in the South Island, 84% (21/25) had CD compared with 57% (13/23) in the North Island (*P* = 0.06).

### *Disease progression*

At the 3‐year follow up, there was progression of Paris classification in 38% (13/34) of children with CD (Table 1) and 25% (2/8) of those initially diagnosed with UC (Table 2). Seven children with CD developed new perianal disease, including four with disease progression limited only to new perianal disease and three who developed perianal disease in addition to other disease progression.

**Table 1:** Age, Paris classification, growth parameters, and disease activity in children with Crohn's disease at diagnosis and 3‐year follow up

		Diagnosis	3‐year follow up
Age in years, median (range)		13.8 (4.7–16.0)	16.7 (7.2–19.0)
Male, *n*/*N* (%)		20/34 (59)	
Paris classification: Age at diagnosis, *n*/*N* (%)	A1a	5/34 (15)	
	A1b	29/34 (85)	
Paris classification: Behavior, *n*/*N* (%)	B1	33/34 (97)	30/34 (88)
	B2		1/34 (3)
	B3	1/34 (3)	3/34 (9)
Paris classification:	L1	2/34 (6)	2/34 (6)
Location, *n*/*N* (%)	L2	12/34 (35)	8/34 (24)
	L3	20/34 (59)	24/34 (71)
Paris classification: Location upper disease, *n*/*N* (%)	L4a	24/34 (71)	22/34 (65)
	L4b	1/34 (3)	1/34 (3)
	L4a and L4b	1/34 (3)	3/34 (9)
Paris classification: Perianal disease, *n*/*N* (%)		5/34 (15)	12/34 (35)
Height z‐score, mean (range), *n*		−0.3 (−4.3 to +2.4), *n* = 32	−0.01 (−2.2 to +2.2), *n* = 31
BMI z‐score, mean (range), *n*		−0.6 (−4.5 to +1.3), *n* = 32	−0.1 (−3.0 to +2.7), *n* = 31
PCDAI, mean (range), *n*		35 (15–68), *n* = 30	6 (0–25), *n* = 30
Modified PCDAI, mean (range), *n*		11 (0–25), *n* = 30	2 (0–13), *n* = 25

BMI, body mass index; PCDAI, pediatric Crohn's disease activity index.

**Table 2:** Age, disease extent, growth parameters, and disease activity in children with ulcerative colitis at diagnosis and 3‐year follow up, including those reclassified as ulcerative colitis (UC) in the intervening 3 years†

		Diagnosis	3‐year follow up
Age in years, median (range)		8.8 (1.0–13.5)	12.3 (4.1–16.8)
Male, *n*/*N* (%)		6/11 (55)	
Paris classification: Extent, *n*/*N* (%)‡	E1	2/11 (18)	1/11 (9)
	E2	2/11 (18)	1/11 (9)
	E3		1/11 (9)
	E4	7/11 (64)	8/11 (73)
Paris classification: Severity, *n*/*N* (%)	S0	10/11 (91)	11/11 (100)
	S1	1/11 (9)	0/11 (0)
Height z‐score, mean (range)		−0.01 (−1.2 to +1.3), *n* = 11	+0.01 (−1.1 to +1.1), *n* = 11
BMI z‐score, mean (range)		+0.3 (−1.7 to 2.3), *n* = 11	+0.6 (−1.5 to +2.2), *n* = 11
PUCAI, mean (range)		44 (30–70), *n* = 11	6 (0–35), *n* = 11

† Including three participants initially diagnosed with IBD‐unclassified (IBDU), who received a diagnosis of UC within the intervening 3 years.

‡ The three participants initially diagnosed with IBDU all were Paris classification extent = E4 at diagnosis and follow up.

BMI, body mass index; PCDAI, pediatric Crohn's disease activity index.

There were no new EIM reported following diagnosis. Of the children with CD, nine had EIM at diagnosis. Three children had rheumatological EIM, including two with polyarthritis and one with previously diagnosed idiopathic juvenile arthritis. Two children had hepatobiliary EIM, including one with primary sclerosing cholangitis (PSC) and one with autoimmune hepatitis and PSC features. Three children had orofacial EIM, and one had erythema nodosum. There was one child with IBDU who had PSC at diagnosis.

### *Growth parameters*

The mean BMI z‐score increased significantly in all patients with IBD (−0.40 to +0.10; *n* = 43, *P* = 0.01) and in the subset of participants with CD (−0.62 to −0.10; *n* = 21, *P* = <0.01). Mean height scores did not change significantly in either all IBD (−0.27 to −0.03; *n* = 43, *P* = 0.14) or CD (−0.33 to −0.10; *n* = 31, *P* = 0.13) patients. The proportion of children with height <third percentile in CD was 13% (4/32) at diagnosis and 3% (1/31) at 3‐year follow up. No child with IBDU or UC had a height <third percentile at diagnosis or follow up.

### *Age differences in IBD characteristics*

There were only five children diagnosed with CD at age <10 years (A1a), comprising one child with terminal ileal (L1), two with colonic (L2), and two with ileocolonic (L3) disease location. Both children with isolated colonic disease at diagnosis progressed to ileocolonic disease by 3‐year follow up. Three children aged <10 years had upper gastrointestinal (UGI) involvement at diagnosis and follow up. All five children diagnosed at age <10 years had inflammatory disease at diagnosis and follow up. While none of these young children had perianal disease at diagnosis, two developed perianal disease within 3 years.

Comparing children diagnosed at age <10 years (A1a) to older children (A1b), 40% (2/5) of younger children compared with 62% (18/29) of older children had ileocolonic disease at diagnosis (*P* = 0.63). Similar proportions of younger and older children had isolated colonic disease at diagnosis (40%, 2/5 *vs* 35%, 10/29).

### *Disease activity*

Comparing diagnosis to 3‐year follow up, there were significant increases in blood hematocrit, hemoglobin, and albumin levels and reductions in blood platelets, erythrocyte sedimentation rate (ESR), C‐reactive protein (CRP), and ferritin levels in all IBD (Table 3). In CD, there were reductions in mean PCDAI and modified PCDAI scores (Table 3). There was also a significant reduction in PUCAI score in the 11 children diagnosed with UC from a mean of 44 at diagnosis to 6 at follow up, *P* = <0.001.

**Table 3:** Inflammatory markers, blood parameters, and disease severity at diagnosis and 3‐year follow up in children diagnosed with inflammatory bowel disease (IBD) in 2015

	All IBD				CD only			
	Mean baseline	Mean follow up	*P*‐value	*n*	Mean baseline	Mean follow up	*P*‐value	*n*
Hct (%)	0.36	0.40	<0.01	36	0.36	0.40	<0.01	29
Hb (g/L)	115	133	<0.01	36	113	135	<0.01	29
Platelets (×10^9^/L)	457	338	<0.01	36	472	334	<0.01	29
Albumin (g/L)	35	39	0.01	34	34	38	0.02	27
ESR (mm/h)	29	11	<0.01	25	32	12	0.01	18
25OH‐VitD (nmol/L)	58	67	0.48	15	55	76	0.12	11
Iron (micromol/L)	8	13	0.03	20	8	13	0.07	16
Ferritin (microgram/L)	76	38	0.03	23	85	40	0.03	19
Transferrin saturation (%)	12%	15%	0.19	21	12%	16%	0.24	17
Transferrin (g/L)	2.4	2.7	0.06	17	2.3	2.7	0.10	14
Vit B12 (pmol/L)	485	372	0.03	13	469	369	0.12	10
PCDAI					35	6	<0.001	28
mod PCDAI					10	2	<0.001	25

	Median baseline (IQR)	Median follow up (IQR)	*P*‐value	*n*	Median baseline (IQR)	Median follow up (IQR)	*P*‐value	*n*
CRP (mg/L)	12 (2–51)	1 (1–6)	0.01	28	13 (5–53)	1 (1–6)	0.03	25

Children without paired data were excluded for each parameter.

25‐OHD, 25‐hydroxyvitamin D; CD, Crohn's disease; CRP, C‐reactive protein; ESR, erythrocyte sedimentation rate; Hb, hemoglobin; Hct, hematocrit; IQR, interquartile range; mod PCDAI, modified PCDAI; PCDAI, pediatric Crohn's disease activity index; PUCAI, pediatric ulcerative colitis activity index; Vit B12, vitamin B12.

### *Disease management*

Most children (91%, 31/34) with CD received EEN at diagnosis. As part of management for a disease flare, six children with CD also received a further course of EEN. Half (*n* = 3) of these subsequent courses were within 2 years of diagnosis, and the remainder were in the third year following diagnosis.

Of all 48 children with IBD, 27 received steroids at a mean of 7 months following diagnosis (range 0–35 months) (Table 4). During the three intervening years, 44% (15/34) of children with CD and 27% (3/11) with UC received biologic therapy. Of the 15 children with CD who received biologic therapy, 11 received infliximab, with 1 subsequently changing to adalimumab, and 4 received adalimumab, with 1 changing to infliximab. Two children with UC changed from infliximab to adalimumab. Three children with CD and one child with UC started biologic therapy within 1 year of diagnosis, and eight (6 CD, 2 UC) started in the second year following diagnosis.

**Table 4:** Disease course and management during 3 years of follow up in children diagnosed with inflammatory bowel disease (IBD) in 2015

	CD, *n* = 34	UC, *n* = 11	IBDU, *n* = 3
Disease flares during 3 years, median (IQR)	1.5 (1–3)	1 (0–3)	1 (0–2)
Hospitalizations during 3 years, median (IQR)	1 (0–2)	1 (0–4)	1 (0–3)
Steroids, % (*n*/*N*)	56% (19/34)	64% (7/11)	33% (1/3)
Cumulative steroid dose†,‡, mean (range), *n*	57 (22–125) mg/kg, *n* = 18	152 (10–379) mg/kg, *n* = 7	250 mg/kg, *n* = 1
Biologic, % (*n*/*N*)	44% (15/34)	27% (3/11)	0
Azathioprine, % (*n*/*N*)	88% (30/34)	45% (5/11)	0
Oral ASA, % (*n*/*N*)	56% (19/34)	100% (11/11)	100% (3/3)
Rectal ASA, % (*n*/*N*)	9% (3/34)	45% (5/11)	0
EEN at diagnosis, % (*n*/*N*)	91% (31/34)	0	0
Received a second course of EEN subsequent to diagnosis, % (*n*/*N*)	18% (6/34)	0	0
Antibiotics at any time, % (*n*/*N*)	56% (19/34)	45% (5/11)	0
Mean total antibiotics courses for 3 years‡, mean (range), *n*	2.8 (1–6), *n* = 19	3.2 (1–7), *n* = 5	0

† Prednisolone equivalents.

‡ Cumulative steroid doses and mean antibiotics per year are of those who received steroids or antibiotics.

ASA, 5‐aminosalicylic acid; CD, Crohn's disease; EEN, exclusive enteral nutrition; IBDU, IBD‐unclassified; IQR, interquartile range; UC, ulcerative colitis.

Of 30 children with CD who received azathioprine, 19 started at <3 months postdiagnosis, 9 commenced at 3–5 months, and 2 children commenced at 6–11 months postdiagnosis. Three children with CD received a second immunomodulatory drug: two received methotrexate and one received 6‐mercaptopurine within 1 year following diagnosis. The five children with UC who received azathioprine started within a year after diagnosis.

The 19 children with CD who required antibiotics as part of IBD management received a mean of 2.8 antibiotic courses, including a mean of 2.3 ciprofloxacin courses and 2.2 metronidazole courses during the 3‐year follow up. Five children with UC received a mean of 3.2 antibiotic courses, including a mean of 2.0 ciprofloxacin courses and 2.0 metronidazole courses during the 3‐year follow up.

At diagnosis, 41% (14/34) of children with CD, 9% (1/11) with UC, and 33% (1/3) with IBDU received treatment for iron deficiency. In the intervening 3 years, 62% (21/34) with CD received iron, including 10 children who received intravenous iron. Of children with UC, 55% (6/11) received iron, including two who received intravenous iron, and one child with IBDU received oral iron.

### *Surgical management*

Of the 34 children with CD, 7 (21%) had undergone a surgical procedure, including five operations for perianal or perivaginal abscess management, one partial colectomy, and one ileal stricturoplasty. Two of the children requiring perianal abscess drainage underwent further surgery; one child had two examinations under anesthesia for perianal pain with no active abscess found, and one child had a revision and then removal of a seton drain. Two children with UC had a colectomy and ileostomy formation, one of whom subsequently required an adhesiolysis for a bowel obstruction.

### *Management at most recent follow up*

At 3 years following diagnosis, 44% of children with CD and 27% with UC were receiving a biologic therapy (Table 5). All children with IBD receiving biologic therapy were also receiving an immunomodulator.

**Table 5:** Current management at most recent follow up 3 years following diagnosis in children diagnosed with inflammatory bowel disease (IBD) in 2015

	CD (*N* = 34), *n* (%)	UC (*N* = 11), *n* (%)	IBDU (*N* = 3) *n* (%)
Oral ASA	12 (35%)	11 (100%)	2 (67%)
Rectal ASA	1 (3%)	0	0
Azathioprine	26 (76%)	45% (5/11)	2 (67%)
Other immunomodulator	2 (6%)	0	0
Biologic (any)	15 (44%)	27% (3/11)	0
Infliximab	11 (32%)	9% (1/11)	0
Adalimumab	4 (12%)	18% (2/11)	0
Steroid (oral)	1 (3%)	0	1 (33%)
Other medication or management	2 EEN	1 Colifoam	
	1 Colifoam		
No management by patient choice	3 (9%)	0	0

ASA, 5‐aminosalicylic acid; CD, Crohn's disease; EEN, exclusive enteral nutrition; IBDU, IBD‐unclassified; UC, ulcerative colitis.

## Discussion

This is the first nationwide cohort reporting the current management and outcomes of childhood IBD in the NZ setting. A striking feature was the high proportion of CD. Although both disease progression and an increase in perianal disease occurred, only three children with CD had penetrating disease at follow up, and one had a stricture suggesting good disease control. Of all IBD patients, more than half received steroids, and some children had substantial exposure.

Childhood IBD has been associated with more severe disease and more extensive location in both CD and UC.^10^, ^11^, ^12^ In childhood CD, ileocolonic location occurs most frequently,^12^, ^17^, ^19^, ^21^ although in young children, isolated colonic disease is also common.^12^, ^17^, ^26^, ^27^, ^28^ Consistent with these studies, most CD was ileocolonic. Although only five children with CD were diagnosed at <10 years, two (40%) of these young children had isolated colonic disease at diagnosis, and both progressed to ileocolonic disease by 3‐year follow up. The proportion of young children with isolated colonic disease was comparable to previous studies, which found isolated colonic disease in 33–41% of young children (defined as <8 to <11 years) diagnosed with CD^12^, ^17^, ^27^ *versus* 6–24% diagnosed as older children (defined as ≥8 to ≥11 years).^12^, ^17^, ^26^, ^27^

The majority of children with CD in the current study had inflammatory disease at diagnosis, consistent with previous studies.^17^, ^21^ Of the children with changes in their Paris classification over time, most had disease extension and only 12% had developed stricturing or penetrating disease at 3‐year follow up. Previous studies have found higher rates of behavior progression, although this was likely contributed to by the longer duration of follow up included in these studies. Two studies of childhood CD identified stricturing or penetrating disease in 58–59% of children at median 7–11 years of follow up.^19^, ^21^ A further study found progression of disease location in 11% and behavior in 29% at 5‐year follow up.^20^ A systematic review reported progression to stricturing disease in 24–43% and to penetrating disease in 14–27% of CD at 4–10 years of follow up.^16^ In the current study, progression of extent occurred in two children with UC. The systematic review also found an increase in pancolitis over time in children with UC.^16^

In this cohort, UGI and perianal disease occurred frequently in CD. On diagnosis, 76% of children with CD had UGI disease, and although no new UGI disease occurred, progression of UGI location occurred in two children. In contrast, two French studies reported lower rates of UGI disease, with rates increasing from 36% at diagnosis to 48% at 7‐year follow up^21^ and from 31% at diagnosis to 42% at 11‐year follow up.^19^ In the current study, perianal disease occurred in 35% of children with CD 3 years following diagnosis. Most studies report lower rates of perianal disease, although there is substantial variation. Two studies reported perianal disease at diagnosis in 9–10% of children,^17^, ^19^ and studies including a systematic review reported perianal disease in 16–30% at 4–11 years following diagnosis.^16^, ^19^, ^20^ However, one Korean study identified perianal fistulae in 50% of CD at 1 year and 55% at 5 years.^14^

Of children with CD, 26% had EIM, comparable to previous studies, which have reported EIM in 20–24% of childhood CD at diagnosis.^17^, ^19^ In addition, a French study found new EIM in 30% of children with CD at 11 years of follow up.^19^

Growth parameters improved during the intervening 3 years. BMI z‐scores significantly increased and were close to the 50th centile at follow up in CD and all IBD. A systematic review of childhood IBD, including studies from 1935 to 2007, found that growth failure, mostly defined as height <third percentile, was more frequent in CD than UC and reported growth failure in CD in 10–56% of children at diagnosis and 10–40% at least 5 years following diagnosis.^16^ In the NZ cohort, 13% of CD had a height <third percentile at diagnosis. Only one child (3%) had height persistently <third percentile at 3‐year follow up, suggesting improved nutrition and good disease control. The same systematic review reported growth failure in 0–10% of children with UC at diagnosis and 0–17% at follow up.^16^ In the NZ cohort, no child with UC or IBDU had height <third centile.

Consistent with this cohort, in which 56% of all children with IBD received steroid management at least once, recent studies demonstrate that many children with CD continue to receive steroids.^14^ A study including children diagnosed with CD from 2006 to 2013 reported that 47% of children received steroids by 1 year and 57% by 5 years following diagnosis.^14^, ^19^, ^21^ Long‐term studies report that 85–96% of children with CD received at least one course of steroids at a median of 7–11 years.^19^, ^21^

Almost half of the children with CD received biologic therapy, consistent with previous studies of childhood CD, which report management with biologic therapy in 11–46% at 1 year^14^ and 24–35% 5–11 years.^14^, ^19^, ^21^ Biologic therapies were prescribed following the NZ pharmacological prescribing step‐up approach requirements.^29^

Most children with CD were managed with EEN at diagnosis, consistent with European Society of Pediatric Gastroenterology, Hepatology and Nutrition (ESPGHAN) and European Crohn's and Colitis Organization (ECCO) guidelines.^30^ Recent studies suggest that the effectiveness of EEN to induce remission following diagnosis in CD is around 80%.^31^, ^32^, ^33^

Although numbers were limited, only 6% of children with CD required intestinal resection within 3 years following diagnosis, which is a lower intestinal surgery rate than previously reported.^14^, ^18^, ^19^, ^20^, ^21^ A systematic review demonstrated a reduction in the 10‐year risk of intestinal surgery over time, from 47 to 38% in CD and from 16 to 14% in UC, comparing people diagnosed in 1956–1970 to diagnoses since 2000.^18^ Studies in children have reported intestinal resection rates in CD of 4.5% at 1 year,^14^ 20% at 3 years,^21^ 17–34% at 5 years,^14^, ^21^ and 43–44% at a median of 11–16 years.^19^, ^20^ Notably, the study reporting an intestinal resection rate at 3 years following diagnosis included children recruited between 1988 and 2002.^21^ This study reported that 24% of patients received infliximab^21^ compared with 44% in our present study, suggesting that intestinal resection rates may have been higher before the widespread use of biologic therapy. The rate of intestinal resection in children with UC 3 years after diagnosis (18%) was similar to the previous studies.^18^

The current study is limited by the number of children included in the cohort, particularly in the subgroups diagnosed with IBDU or UC. Overall outcomes were analyzed because sample size limited analysis of outcomes by intervention. However, this is the only study reporting disease course in NZ children with IBD and included all children known to be diagnosed with IBD in 2015. Furthermore, this follow‐up study was able to report on the outcomes of 94% of the original cohort. Although these data provide perspective on the medical, growth, and disease factors, they do not provide an understanding of the wider impact of the disease, such as social and educational outcomes.

In conclusion, most NZ children with IBD are in remission 3 years following diagnosis with improved disease activity scores and growth. Although some children had progression of disease, this was mostly limited to progression of location, and only a small number of children with CD had noninflammatory disease at follow up. Almost half of children with CD received a biologic medication, and approximately half of all children with IBD received steroids at some time during the three intervening years. These data establish a perspective on the short‐term outcomes of NZ children diagnosed with IBD and illustrate the role of nutritional and biologic therapies.
